# Supplementary material for: Altered extracellular matrix structure and elevated stiffness in a brain organoid model for disease
Source: Nat Commun. 2025 May 1;16:4094. doi: 10.1038/s41467-025-59252-w (PMC12045990; doi:10.1038/s41467-025-59252-w)
Supplement: Supplementary file 1 — Supplementary Information [file 41467_2025_59252_MOESM1_ESM.pdf]

Supplementary figures

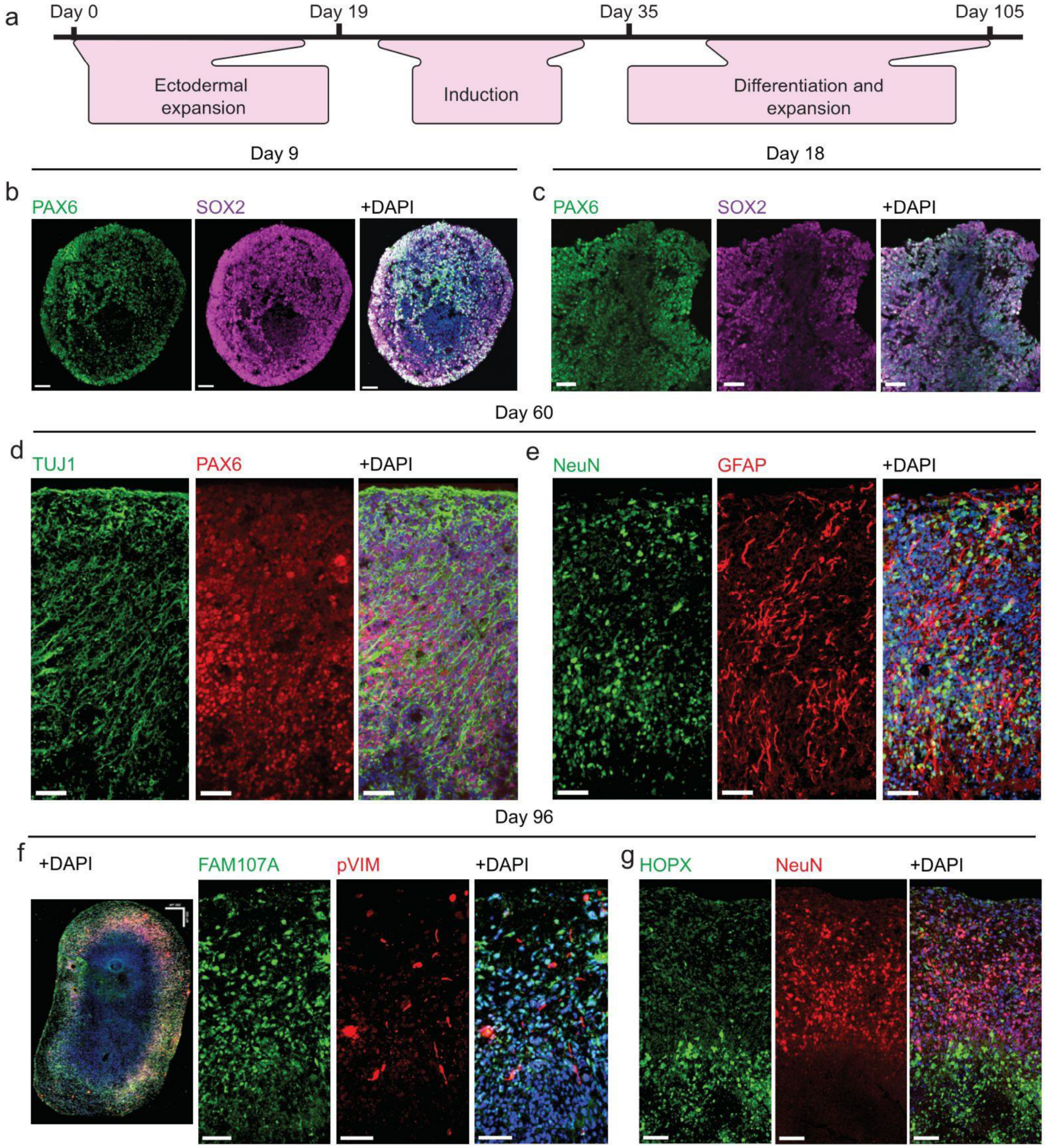

Supplementary Fig. S1: Cortical Organoids (CorticOs). a, Timeline of corticOs development protocol. **b-d,** Proliferative markers SOX2 and PAX6 in ectodermal-like organoids after 9 days (**b**) as well as on day 18 (**c**). **d-e,** PAX6^{+} cells were still prevalent on day 60, along with neuronal processes marker TUJ1 (**d**), NeuN^{+} neurons, and GFAP^{+} astrocytes (**e**). **f-g,** On day 96, FAM107^{+} and pVIM^{+} (**f**), HOPX^{+} basal radial glia cells were expressed, and NeuN^{+} neurons (**g**). All figures have a scale bar of 50 µm.

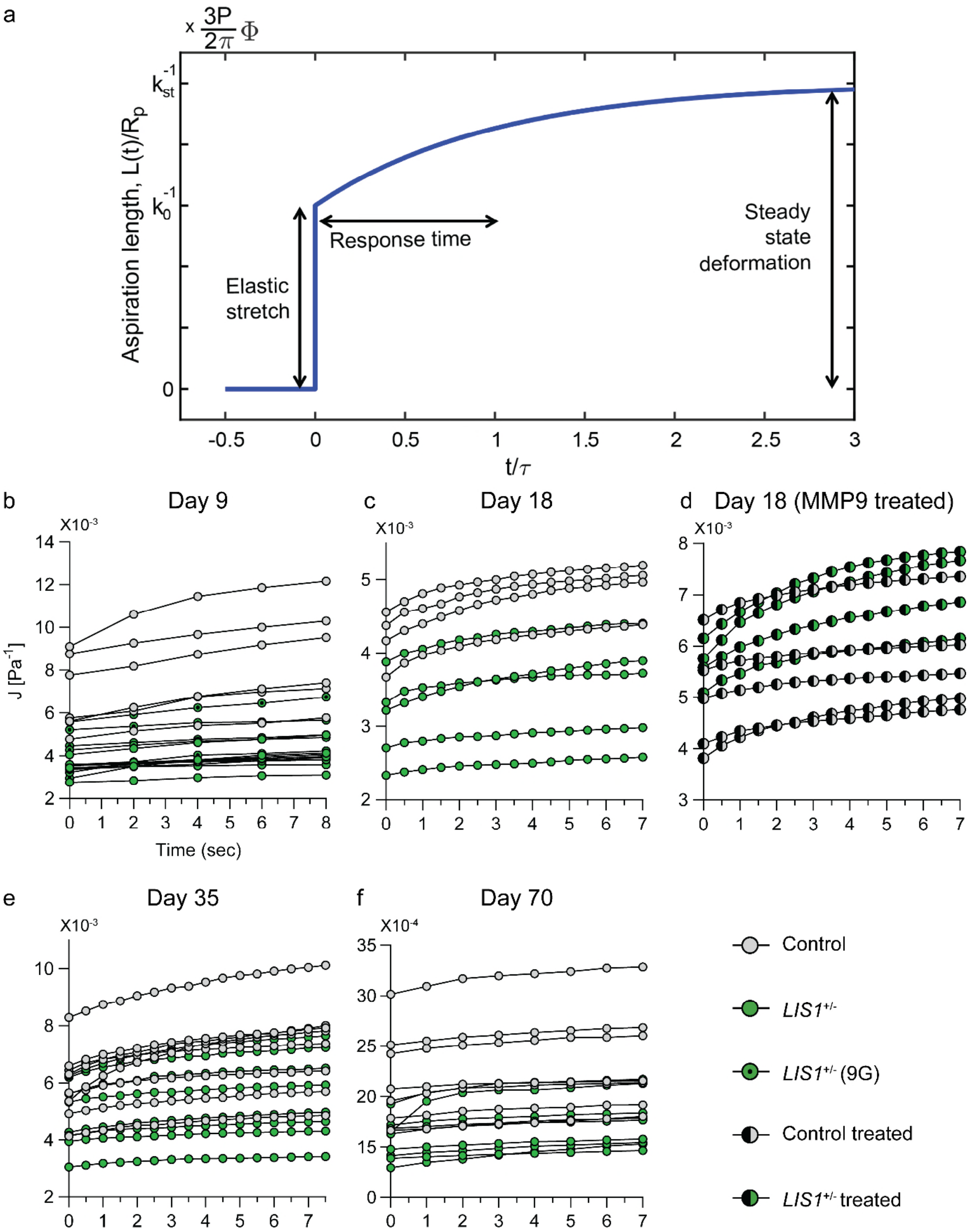

Supplementary Fig. S2: Creep test MPA rheology. a, The aspiration length $L(t)$ of an SLS material in a creep test. The material stretches elastically to an aspirated length that is proportional to ${k_0}^{-1}$, then aspiration continuous over a length scale τ that marks viscoelastic deformation before approaching a steady-state deformation with aspiration length that is proportional to ${k_{st}}^{-1}$. $k_0 = k_1 + k_2$, $k_{st} = k_1$ and $\tau = \frac{\mu \cdot (k_1 + k_2)}{(k_1 k_2)}$ are the viscoelastic elements of the SLS

material (**Fig. 1**). **b-f,** Creep test measurements of Day-9, 18, 35 and 70 organoids per the specified treatments. One measurement was performed for each organoid. Unless specified otherwise, "treated" refers to MMP9 treatment. The analysis included: Day-9, $N_{WT} = 6$, $N_{10F} = 8$, $N_{9G} = 7$. Day-18, $N_{WT} = 4$, $N_{10F} = 5$, Day-35, $N_{WT} = 8$, $N_{10F} = 8$, and Day-70, $N_{WT} = 7$, $N_{10F} = 9$.

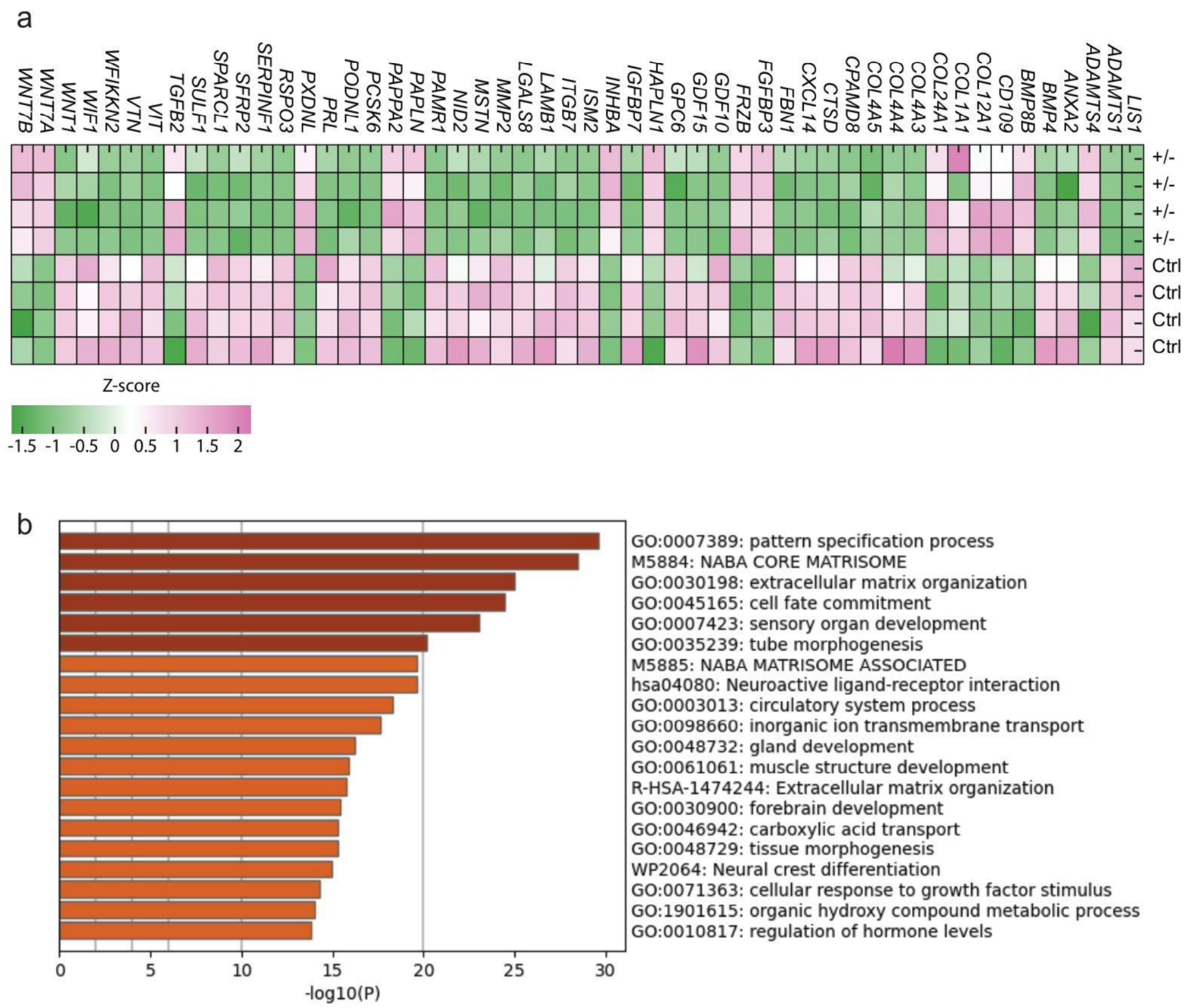

Supplementary Fig. S3: RNA-seq of 105 days old corticOs reveal altered ECM expression. a, Heatmap of top DE mRNAs between control and *LIS1*$^{+/-}$ organoids, after logarithmic transformation of counts and z-score normalisation*. **b,** Metascape analysis of 2064 genes that were DE between control and *LIS1*$^{+/-}$mutant organoids. The analysis included: $N_{corticOs} = 4$; $n_{corticOs} = 6\text{-}8$.

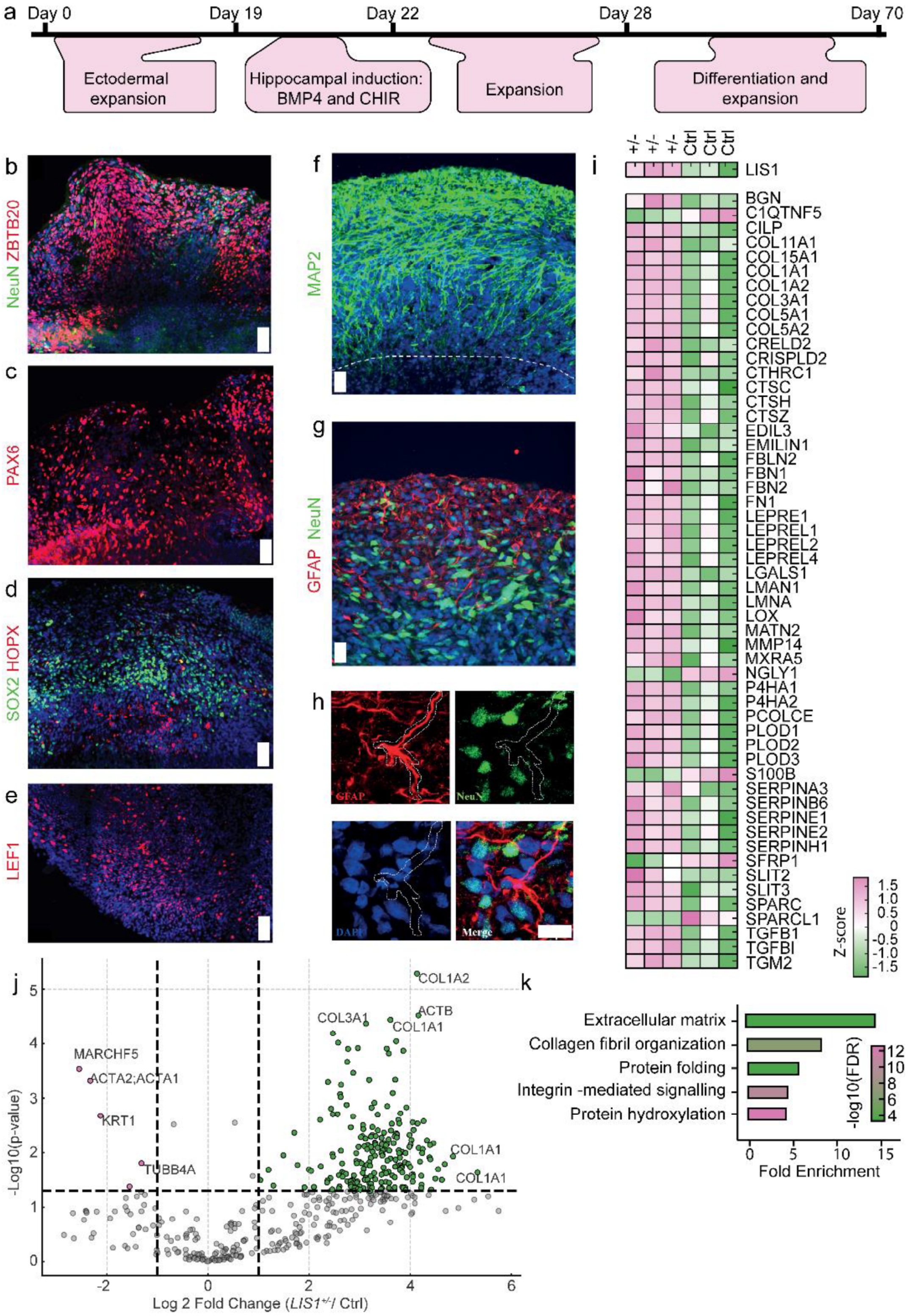

Supplementary Fig. S4: Hippocampal organoids characterization and comparative proteomics of *LIS1*$^{+/-}$ and control samples. a, Timeline of hippOs development protocol. **b-e,** 70 days old hippOs expressed the post-mitotic neurons marker NeuN and the dentate gyrus marker ZBTB20 (**b**), the progenitor markers PAX6 (**c**) HOPX, and SOX2

(**d**), as well as the transcription factor LEF1. **f-h**, hippOs also expressed MAP2$^+$ neuronal processes markers, (**f**) GFAP$^+$ astrocytes, and NeuN$^+$ neuronal cells, and (**g**) shows a close-up visualization of these markers. **i**, Heatmap of DE matrisomal proteins in *LIS1*$^{+/-}$ and control hippOs. Cell color expresses normalized reads following logarithmic transformation and Z-score normalization*. **j,** Volcano plot of hydroxylation in the MS peptide reads. Points marked in green and pink show the top enriched PTM targets in the *LIS1*$^{+/-}$ and control organoids, respectively. **k,** False discovery rate of DE proteins following a WebGestalt Gene Set Enrichment Analysis (GSEA) analysis [78]. Scale bars in **b-e** demonstrate 50 µm and 10 µm in **f-h**. The mass spectrometry included $N_{hippOs} = 3$; $n_{hippOs} = 5\text{-}8$.

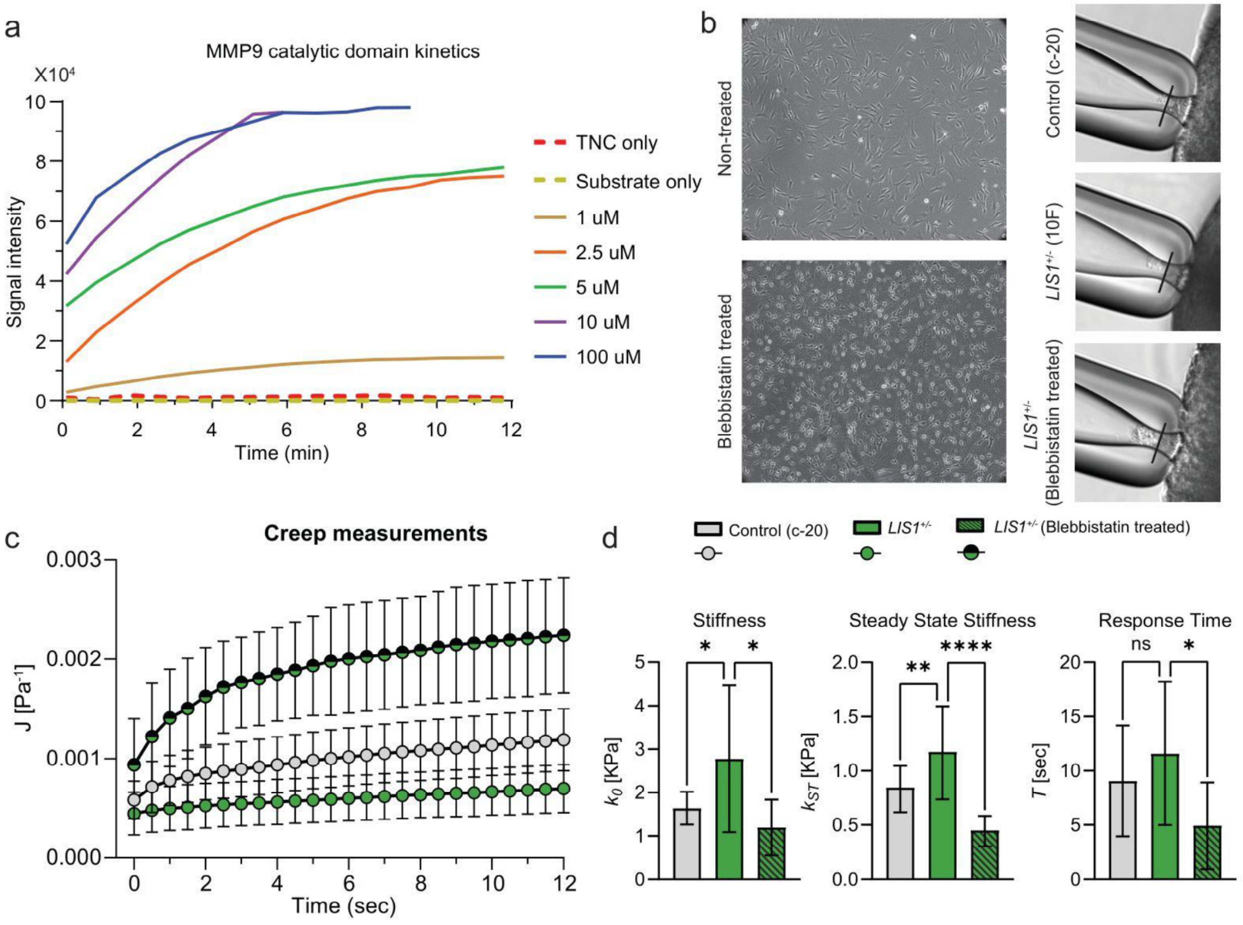

Supplementary Fig. S5: a, MMP9 catalytic domain ELISA activity assay and blebbistatin treatment results. Fluorescence signal intensity at uniform time intervals for two control conditions - 'TNC-only' and 'TNC + substrate' and experimental conditions with 20 nM of MMP9 catalytic domain and varying concentrations of substrate (from 1 µM to 100 µM). The calculated kinetics parameters are: V_m = (185±33) uM/s and K_m = (2±1) uM. **b-d, Cell-scale MPA confirms *LIS1*-mutation organoid stiffening.** (**b**) Representative images of human telomerase-positive skin fibroblasts that were treated with 50 uM Blebbistatin for two hours and compared with non-treated cells. Cytoplasmic blebbing marks Myosin-II ATPase inhibition. Representative aspiration snapshots of Day-18 control, *LIS1*$^{+/-}$(10F) and blebbistatin-treated *LIS1*$^{+/-}$(10F) organoids. Snapshots were recorded after 4 sec under 1.3±0.2 kPa suction. Pipette inner radius: 12.5 mm **(c)** Creep compliance and SLS fits of (i) control, (ii) *LIS1*$^{+/-}$, and (iii) blebbistatin-treated *LIS1*$^{+/-}$ organoids represent multiple sites that were measured on nControl = 6, n*LIS1+/-* = 6, n*LIS1+/-*-(Blebb) = 4 organoids per condition. **(d)** Fitted instantaneous stiffness, steady-state stiffness and response time are compared between conditions (One-Way ANOVA test N = 5, α = 0.05).

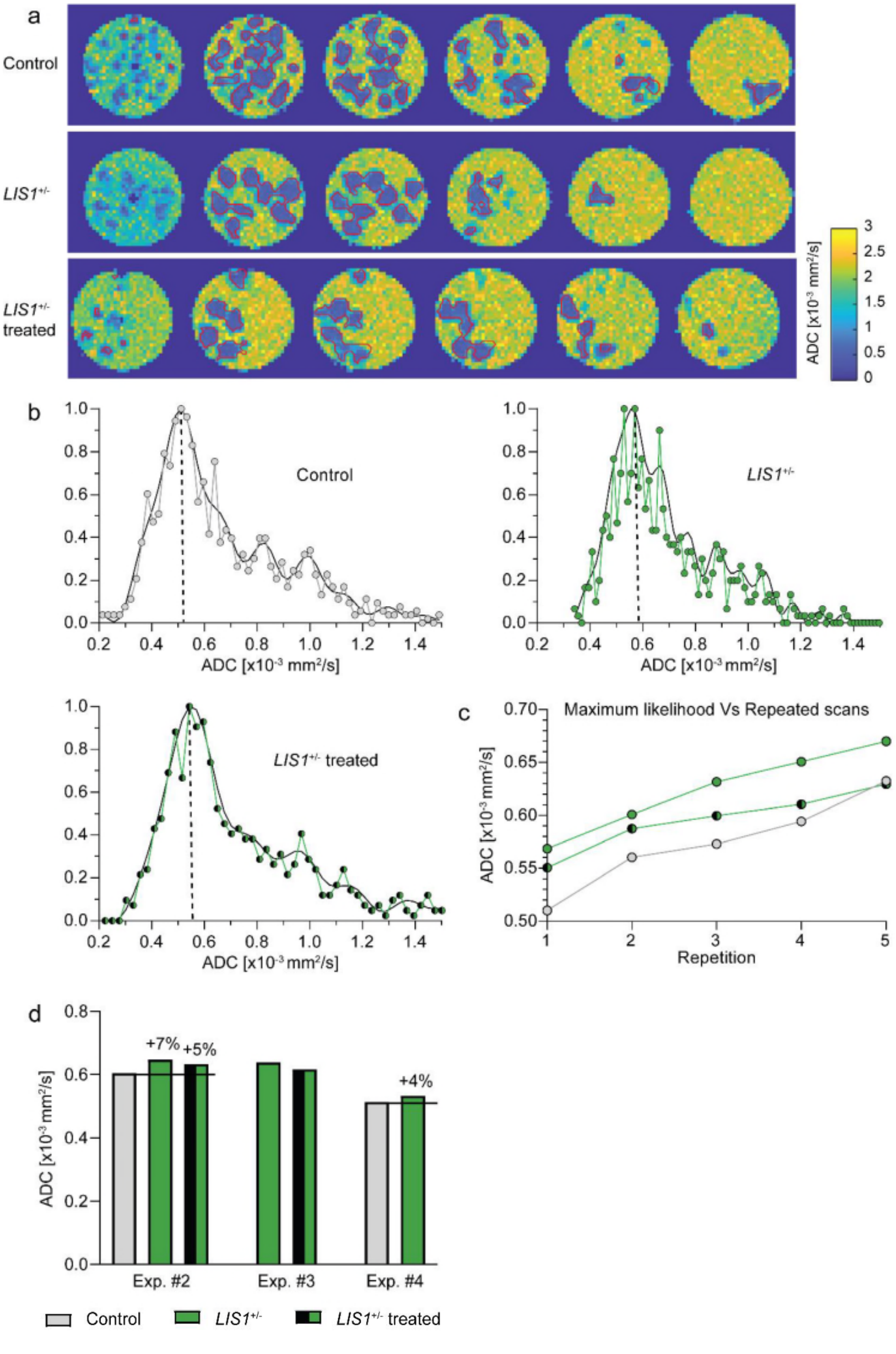

Supplementary Fig. S6: MRI additional information. a, ADC maps including an overlay of identifying contours of control, *LIS1*$^{+/-}$ and MMP9-treated *LIS1*$^{+/-}$ corticOs in Exp.#1 (from top to bottom). Number of voxels for each group was 942, 618,627, respectively. **b,** ADC distribution and the estimated maximum likelihood position from a single scan in Exp.#1 (shown for all three groups). **c,** Estimated maximal likelihood position of the ADC as function of the repeated scans in Exp.#1. **d,** Estimated ADC maximum likelihood positions in three additional experiments (Exp.#2-4). An average number of voxels for each experiment was: 966, 3925, 3176.

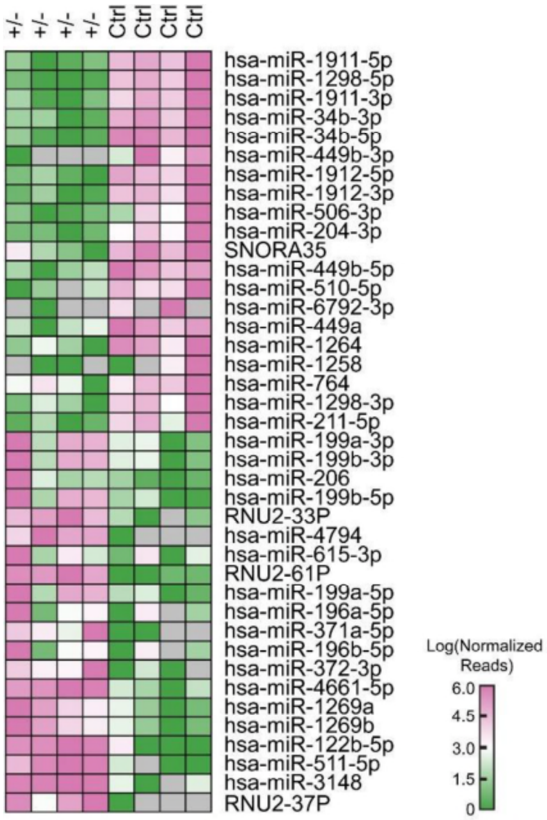

Supplementary Fig. S7: miRNA-seq of 105 days old corticOs. Heatmap of top miRNAs DE between control and *LIS1*$^{+/-}$ organoids. The scale for the heatmap represents normalised reads on logarithmic scale. $N_{corticOs} = 4$; $n_{corticOs} = 6\text{-}8$.

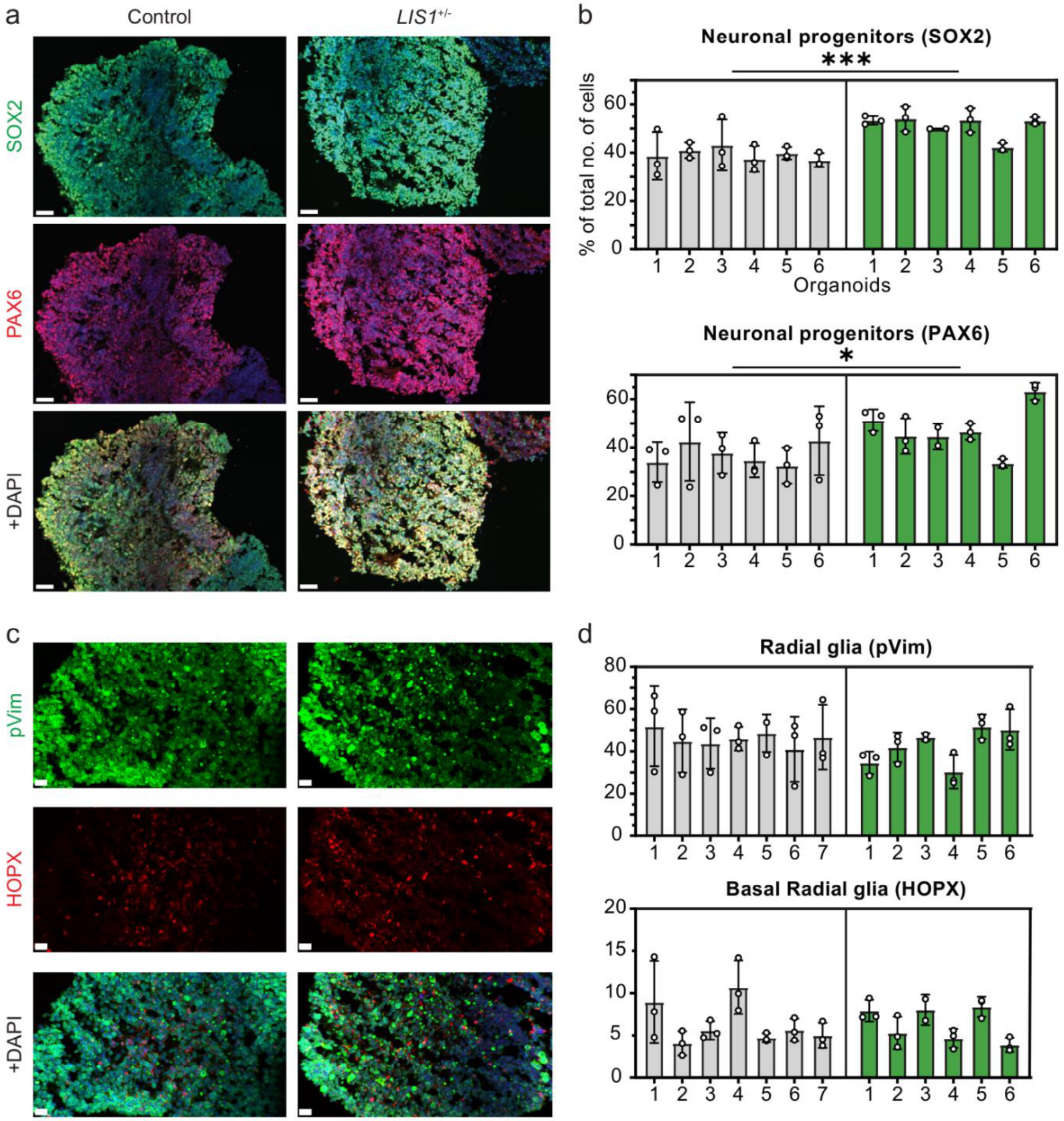

Supplementary Fig. S8: Progenitors and neurons in corticOs. Immunohistochemistry of 18 days old control and *LIS1*$^{+/-}$ organoids showing **(a)** neural progenitor markers SOX2 and PAX6, and **(c)** basal radial glia marker pVim and HOPX. **b,** Percentage of the total number of cells that are progenitors (SOX2^{+}, PAX6^{+}), and **(d)** pVim^{+} and HOPX^{+} cells on day 18 (Nested One-Way ANOVA, n = 7, N = 3, α = 0.05). Images scale bar represents 50 µm.

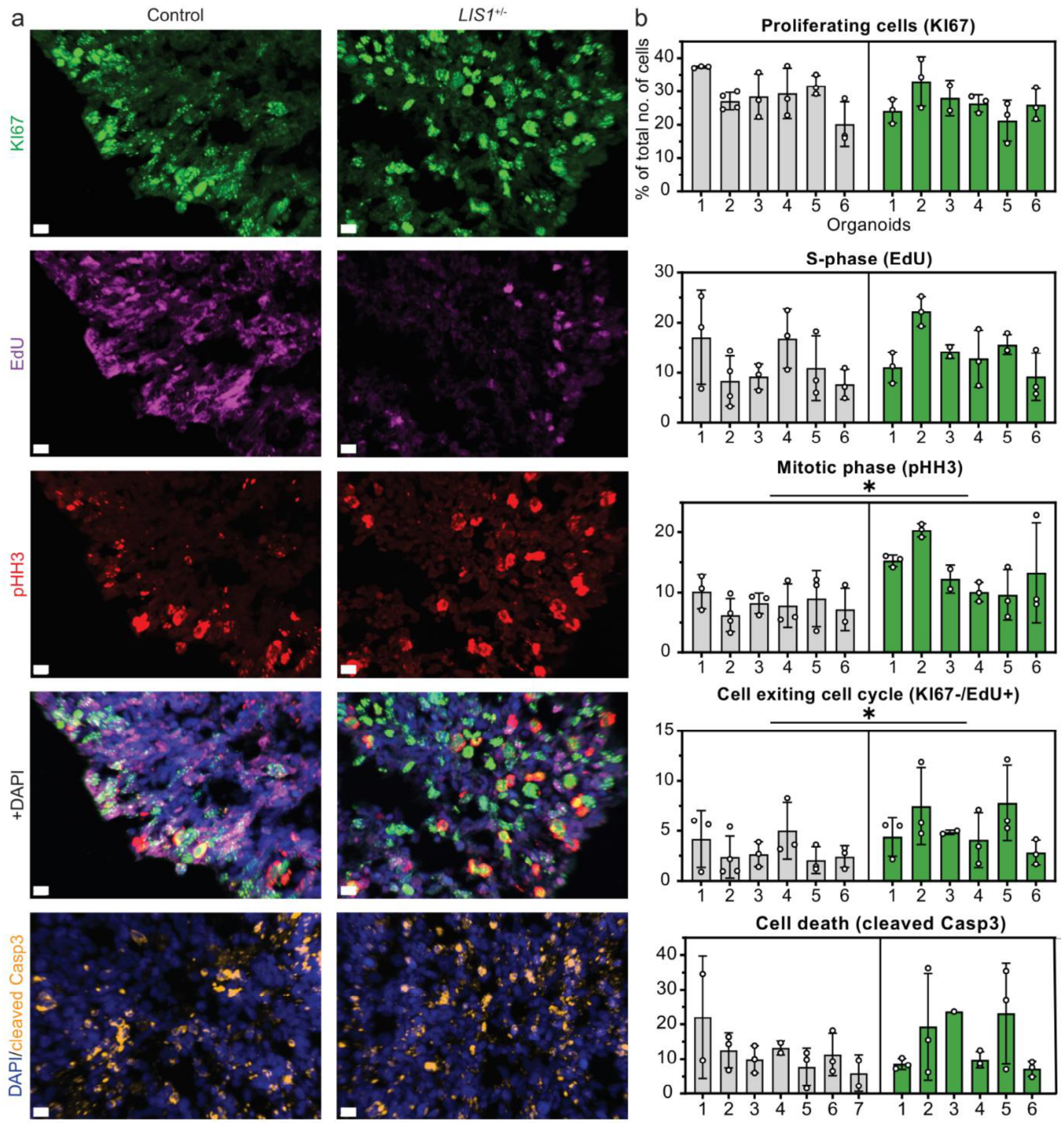

Supplementary Fig. S9: **Proliferation and cell death in corticOs. a,** Immunohistochemistry of 18 days old control and *LIS1*$^{+/-}$ organoids showing the proliferation markers KI67, EdU, pHH3, and cell death marker cleaved Casp3. **b,** Percentage of the total number of cells that are proliferative (KI67^{+}), in initial S-phase (EdU^{+}), mitotically active (pHH3^{+}) and undergoing cell death (cleaved Casp3^{+}) respectively, in day 18 control and *LIS1*$^{+/-}$ organoids. Nested One-Way ANOVA, $n = 7$, $\alpha = 0.05$. Scale bar of images represents 10 μm.

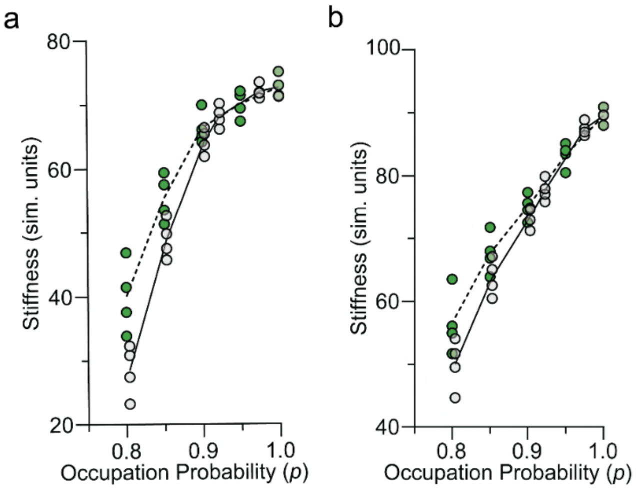

Supplementary Fig. S10: Computational model scenarios of stiffness prediction. a, Same plot as in **Fig. 7i** but with $R=1$ by decreasing k_s by a factor of 10, resulting in a smaller stiffness. **b,** Same plot as in the previous supplement figure with an increased number of cells ($N_C=42$). With the decreased R, we did not find as significant a difference in stiffness between the control and mutant cases as in (**Fig. 7i)**. However, the trend of a larger R (**Fig. 7i**) is more in line with stiffer collagen fibers. Moreover, by increasing the number of cells, the mechanics become less dominated by the fibers and more by the cells, the former of which is organized similarly between the two cases, hence, more similar stiffnesses even for smaller occupation probabilities.

Supplementary Table 1 : List of Antibodies

Application	Target	Species	Dilution	Company	Catalog number
IHC	Collagen type 3 α-chain 1	Rabbit	1:200	Abcam	ab7778
IHC	Collagen type 4 α-chain 1	Rabbit	1:500	Abcam	ab236640
IHC	FAM107A	Rabbit	1:250	Sigma	HPA055888
IHC	GFAP	Rabbit	1:2000	DAKO	Z033401-2
IHC	HOPX	Rabbit	1:250	Sigma	HPA030180
IHC	HOPX	Mouse	1:50	Santa Cruz	398703
IHC	LEF1	Rabbit	1:200	Cell signalling	2230
IHC	NeuN	Mouse	1:200	Millipore	MAB377
IHC	PAX6	Chicken	1:250	DSHB	AB528427
IHC	PAX6	Rabbit	1:100	BioLegend	901301
IHC	pVIM	Mouse	1:100	MBL	D076-3
IHC	SOX2	Mouse	1:300	Santa Cruz	sc-365823
IHC	TUJ1	Mouse	1:200	Convance	Ab18207
IHC	ZBTB20	Rabbit	1:200	Sigma	HPA016815
IHC	MAP2	Mouse	1:200	Sigma	M9940
IHC	KI67	Mouse	1:400	BD Pharmingen	550609
IHC	pHH3	Rabbit	1:200	MilliporeSigma	06-570
IHC	cleaved Caspase3	Rabbit	1:200	Cell signaling	9661
WB	LMNB1	Rabbit	1:10,000	Abcam	ab16048
WB	LMNA/C	Mouse	1:5,000	Cell signalling	4777
WB	γH2AX	Rabbit	1:50	R&D systems	AF2288
WB	GAPDH	Rabbit	1:10,000	Cell Signalling	2118
WB	ACTIN	Mouse	1:10,000	Sigma	a5441
