# Supplementary material for: Altered extracellular matrix structure and elevated stiffness in a brain organoid model for disease
Source: Nat Commun. 2025 May 1;16:4094. doi: 10.1038/s41467-025-59252-w (PMC12045990; doi:10.1038/s41467-025-59252-w)
Supplement: Supplementary file 2 — Description of Additional Supplementary Files [file 41467_2025_59252_MOESM2_ESM.pdf]

Description of Additional Supplementary Files

Supplementary Data 1: Proteomics of cortical organoids (CorticO) day 35 (D35).

Supplementary Data 2: Proteomics of cortical organoids (CorticO).

Supplementary Data 3: RNA-seq of cortical organoids (CorticO).

Supplementary Data 4: Proteomics of hippocampal organoids (HippOs) day 70 (D70).

Supplementary Data 5: Post-translational modifications PTM MetaMorpheus.

Supplementary Data 6: RNA-seq analysis of rescue experiment.

Supplementary Data 7: Small RNA-seq of cortical organoids (CorticO) day 105 (D105).

Supplementary Data 8: Analysis of microRNA (mIR) pathways.
