# Supplementary material for: Altered extracellular matrix structure and elevated stiffness in a brain organoid model for disease
Source: Nat Commun. 2025 May 1;16:4094. doi: 10.1038/s41467-025-59252-w (PMC12045990; doi:10.1038/s41467-025-59252-w)
Supplement: Supplementary file 11 — Reporting Summary [file 41467_2025_59252_MOESM11_ESM.pdf]

nature portfolio

Corresponding author(s): Professor Orly Reiner

Last updated by author(s): YYYY-MM-DD

Reporting Summary

Nature Portfolio wishes to improve the reproducibility of the work that we publish. This form provides structure for consistency and transparency in reporting. For further information on Nature Portfolio policies, see our Editorial Policies and the Editorial Policy Checklist.

Statistics

For all statistical analyses, confirm that the following items are present in the figure legend, table legend, main text, or Methods section.

n/a	Confirmed	
☐	☒	The exact sample size (*n*) for each experimental group/condition, given as a discrete number and unit of measurement
☐	☒	A statement on whether measurements were taken from distinct samples or whether the same sample was measured repeatedly
☐	☒	The statistical test(s) used AND whether they are one- or two-sided<br>*Only common tests should be described solely by name; describe more complex techniques in the Methods section.*
☒	☐	A description of all covariates tested
☐	☒	A description of any assumptions or corrections, such as tests of normality and adjustment for multiple comparisons
☐	☒	A full description of the statistical parameters including central tendency (e.g. means) or other basic estimates (e.g. regression coefficient) AND variation (e.g. standard deviation) or associated estimates of uncertainty (e.g. confidence intervals)
☐	☒	For null hypothesis testing, the test statistic (e.g. *F*, *t*, *r*) with confidence intervals, effect sizes, degrees of freedom and *P* value noted<br>*Give P values as exact values whenever suitable.*
☒	☐	For Bayesian analysis, information on the choice of priors and Markov chain Monte Carlo settings
☒	☐	For hierarchical and complex designs, identification of the appropriate level for tests and full reporting of outcomes
☒	☐	Estimates of effect sizes (e.g. Cohen's *d*, Pearson's *r*), indicating how they were calculated

Our web collection on statistics for biologists contains articles on many of the points above.

Software and code

Policy information about availability of computer code

Data collection	The computational modeling code related to this manuscript is deposited in Zenodo repository and available for public access in: https://doi.org/10.5281/zenodo.15028450
Data analysis	All statistical analysis have been performed with GraphPad Prism 10.4.1

For manuscripts utilizing custom algorithms or software that are central to the research but not yet described in published literature, software must be made available to editors and reviewers. We strongly encourage code deposition in a community repository (e.g. GitHub). See the Nature Portfolio guidelines for submitting code & software for further information.

Data

Policy information about availability of data

All manuscripts must include a data availability statement. This statement should provide the following information, where applicable:

- Accession codes, unique identifiers, or web links for publicly available datasets
- A description of any restrictions on data availability
- For clinical datasets or third party data, please ensure that the statement adheres to our policy

The processed RNA-seq data generated in this study have been deposited in the GEO database under accession code GSE228926 (https://www.ncbi.nlm.nih.gov/geo/query/acc.cgi?acc=GSE228926).

The mass spectrometry proteomics data generated in this study have been deposited in the ProteomeXchange Consortium96 via the PRIDE97 partner repository with the dataset identifier PXD040813 (cortical organoids), PXD040911 (hippocampal organoids), and PXD049178 (early organoids).

Research involving human participants, their data, or biological material

Policy information about studies with human participants or human data. See also policy information about sex, gender (identity/presentation), and sexual orientation and race, ethnicity and racism.

Reporting on sex and gender	Regarding LIS1 mutations, no sex- or gender-based analyses were performed because the phenotype associated with these mutations is exceptionally severe and does not exhibit gender bias. The stem cells used in this study are female (46,XY), and the human brain organoids derived from these cells demonstrate a clear and noticeable phenotype, allowing for robust analysis of the mutation's effects without the need for sex-based differentiation.
Reporting on race, ethnicity, or other socially relevant groupings	*Please specify the socially constructed or socially relevant categorization variable(s) used in your manuscript and explain why they were used. Please note that such variables should not be used as proxies for other socially constructed/relevant variables (for example, race or ethnicity should not be used as a proxy for socioeconomic status). Provide clear definitions of the relevant terms used, how they were provided (by the participants/respondents, the researchers, or third parties), and the method(s) used to classify people into the different categories (e.g. self-report, census or administrative data, social media data, etc.) Please provide details about how you controlled for confounding variables in your analyses.*
Population characteristics	*Describe the covariate-relevant population characteristics of the human research participants (e.g. age, genotypic information, past and current diagnosis and treatment categories). If you filled out the behavioural & social sciences study design questions and have nothing to add here, write "See above."*
Recruitment	*Describe how participants were recruited. Outline any potential self-selection bias or other biases that may be present and how these are likely to impact results.*
Ethics oversight	*Identify the organization(s) that approved the study protocol.*

Note that full information on the approval of the study protocol must also be provided in the manuscript.

Field-specific reporting

Please select the one below that is the best fit for your research. If you are not sure, read the appropriate sections before making your selection.

☒ Life sciences ☐ Behavioural & social sciences ☐ Ecological, evolutionary & environmental sciences

For a reference copy of the document with all sections, see nature.com/documents/nr-reporting-summary-flat.pdf

Life sciences study design

All studies must disclose on these points even when the disclosure is negative.

Sample size	Biological replicates were chosen more than equal to 5 brain organoids per cell line and at least 3 technical replicates were performed in each experiment to take in consideration the sample variability.
Data exclusions	Data points excluded were based on outliers and have been mentioned in the data supporting the graphs in figures
Replication	The experiments were replicated and the data obtained was reproducible.
Randomization	The organoids were chosen randomly from the culture dishes and grouped into replicates for the rheology and high-throughput omics experiments
Blinding	Blinding was not relevant to our study

Reporting for specific materials, systems and methods

We require information from authors about some types of materials, experimental systems and methods used in many studies. Here, indicate whether each material, system or method listed is relevant to your study. If you are not sure if a list item applies to your research, read the appropriate section before selecting a response.

Materials & experimental systems

n/a	Involved in the study	
☐	☒	Antibodies
☐	☒	Eukaryotic cell lines
☒	☐	Palaeontology and archaeology
☒	☐	Animals and other organisms
☒	☐	Clinical data
☒	☐	Dual use research of concern
☒	☐	Plants

Methods

n/a	Involved in the study	
☒	☐	ChIP-seq
☒	☐	Flow cytometry
☐	☒	MRI-based neuroimaging

Antibodies

Antibodies used

IHC Collagen type 3 α-chain 1 Abcam ab7778
IHC Collagen type 4 α-chain 1 Abcam ab236640
IHC FAM107A Sigma HPA055888
IHC GFAP DAKO Z033401-2
IHC HOPX Sigma HPA030180
IHC HOPX Santa Cruz 398703
IHC LEF1 Cell signalling 2230
IHC NeuN Millipore MAB377
IHC PAX6 DSHB AB528427
IHC PAX6 BioLegend 901301
IHC pVIM MBL D076-3
IHC SOX2 Santa Cruz sc-365823
IHC TUJ1 Convance Ab18207
IHC ZBTB20 Sigma HPA016815
IHC MAP2 Sigma M9940
IHC KI67 BD Pharmingen 550609
IHC pHH3 MilliporeSigma 06-570
IHC cleaved Caspase3 Cell signaling 9661
WB LMNB1 Abcam ab16048
WB LMNA/C Cell signalling 4777
WB γH2AX R&D systems AF2288
WB GAPDH Cell Signalling 2118
WB ACTIN Sigma a5441

Validation

Describe the validation of each primary antibody for the species and application, noting any validation statements on the manufacturer's website, relevant citations, antibody profiles in online databases, or data provided in the manuscript.

Eukaryotic cell lines

Policy information about cell lines and Sex and Gender in Research

Cell line source(s)

An NIH-approved human embryonic stem cell (ESC) line NIHhESC-10-0079, WIBR3 (W3), was used in this study. Isogenic mutant cell-line clones were previously generated by CRISPR-Cas9 mediated heterozygous deletion in the LIS1 gene. The pX335 plasmid, an empty Cas9 nickase plasmid used in creating the original LIS1+/- cell line, was electroporated into the parental WIBR3 line to produce a second control. Finally, a single colony was isolated from the WIBR3 line through sub-cloning and was used as an additional control

Authentication

The control WIBR3 stem cell line is routinely cultured and checked for pluripotency. The LIS1 mutant cell line was validated by sequencing and the other clones were obtained by antibiotic selection and sub-cloning.

Mycoplasma contamination

All lines tested negative for Mycoplasma contamination

Commonly misidentified lines
(See ICLAC register)

Name any commonly misidentified cell lines used in the study and provide a rationale for their use.

Plants

Seed stocks

Report on the source of all seed stocks or other plant material used. If applicable, state the seed stock centre and catalogue number. If plant specimens were collected from the field, describe the collection location, date and sampling procedures.

Novel plant genotypes

Describe the methods by which all novel plant genotypes were produced. This includes those generated by transgenic approaches, gene editing, chemical/radiation-based mutagenesis and hybridization. For transgenic lines, describe the transformation method, the number of independent lines analyzed and the generation upon which experiments were performed. For gene-edited lines, describe the editor used, the endogenous sequence targeted for editing, the targeting guide RNA sequence (if applicable) and how the editor was applied.

Authentication

Describe any authentication procedures for each seed stock used or novel genotype generated. Describe any experiments used to assess the effect of a mutation and, where applicable, how potential secondary effects (e.g. second site T-DNA insertions, mosiacism, off-target gene editing) were examined.

Magnetic resonance imaging

Experimental design

Design type

No functional MRI

Design specifications

NA, no task or resting state analysis

Behavioral performance measures

NA, no task, resting state or behavior analysis

Acquisition

Imaging type(s)

Diffusion Weigthed MRI

Field strength

15.2 T

Sequence & imaging parameters

spin-echo scan with diffusion weighting was performed. Four experiments were performed with the following parameters: Exp.#1: TR/TE 400/13.7 ms, FOV 12x12 mm2, in-plane resolution 100x100 µ2, slice thickness 200 µ, 20 slices, ten averages, scan duration 48 minutes. The scan was repeated five times. Exp. #2: TR/TE 400/13.7 ms, FOV 18x12 mm2, in-plane resolution 100x100 µ2, slice thickness 200 µ, nine slices, ten averages, scan duration 48 minutes. Exp.#3: TR/TE 400/13.7 ms, FOV 12x12 mm2, in-plane resolution 100x100 µ2, slice thickness 200 µ, 20 slices, 20 averages, scan duration 1h36m0s0ms. Exp.#4: TR/TE 300/12.7 ms, FOV 12.6x2.4 mm2, in-plane resolution 100x100 µ2, slice thickness 100 µ, 60 slices (3D acquisition), one averages, scan duration 1h12m0s0ms.

Area of acquisition

The area of acquisition was chosen to include all brain organoids in the field-of-view.

Diffusion MRI ☒ Used ☐ Not used

Parameters

Exp.#1: The scan included six b-values - 0, 200, 400, 600, 1000, 1200 s/mm2. Exp. #2: The scan included six bvalues - 0, 200, 400, 600, 1000, 1200 s/mm2. Exp.#3: The scan included six bvalues - 0, 200, 400, 600, 1000, 1200 s/mm2. Exp.#4: The scan included four b-values - 0, 200,600,1000 s/mm2.

Preprocessing

Preprocessing software

Matlab 2021a scripts were used. The Diffusion Weighted Imaging (DWI) MRI dataset was collected to assess the differences between brain-organoid groups. Based on the collected dataset, the Aperient Diffusion Coefficient (ADC) maps were calculated using a monoexponential fit. The images with the highest b-value (the degree of diffusion weighting) were used to segment and identify the voxels of the brain organoid tissue. Supplementary Fig. S5a shows the ADC maps and the contour of the segmented voxels. A normalized distribution of the ADC values in the identified voxels for each type, consisting of 100 bins, was defined. A denoising of the distribution profile was then performed, removing high-frequency components using FT. The distribution of the diffusion in the brain organoids tissue results in an asymmetric profile; therefore, the maximal likelihood position was defined as a centre of points with 2/3Imax. intensity (Imax was found based on the denoised distribution and the two points with 2/3Imax intensity from both sides of the distribution by interpolation). The same steps were repeated for each organoid type and scan.

Normalization

A normalized distribution of the ADC values in the identified voxels for each type, consisting of 100 bins, was defined.

Normalization template

Describe the template used for normalization/transformation, specifying subject space or group standardized space (e.g. original Talairach, MNI305, ICBM152) OR indicate that the data were not normalized.

Noise and artifact removal

A denoising of the distribution profile was then performed, removing high-frequency components using FT.

Volume censoring

The images with the highest b-value (the degree of diffusion weighting) were used to segment and identify the voxels of the brain organoid tissue. This method was chosen, since this allowed for clear segmentation between the organoids and the

surrounding background.

Statistical modeling & inference

Model type and settings: NA, no task or resting state analysis

Effect(s) tested: NA, no task or resting state analysis

Specify type of analysis: ☐ Whole brain ☐ ROI-based ☐ Both

Statistic type for inference (See Eklund et al. 2016): *Specify voxel-wise or cluster-wise and report all relevant parameters for cluster-wise methods.*

Correction: *Describe the type of correction and how it is obtained for multiple comparisons (e.g. FWE, FDR, permutation or Monte Carlo).*

Models & analysis

n/a	Involved in the study
☒	☐ Functional and/or effective connectivity
☒	☐ Graph analysis
☒	☐ Multivariate modeling or predictive analysis
